# The influence of allogenic blood transfusion in patients having free-flap primary surgery for oral and oropharyngeal squamous cell carcinoma

**DOI:** 10.1038/sj.bjc.6603013

**Published:** 2006-03-07

**Authors:** T Szakmany, M Dodd, G A Dempsey, D Lowe, J S Brown, E D Vaughan, S N Rogers

**Affiliations:** 1 Intensive Care Unit, University Hospital Aintree, Liverpool L9 1AL, UK; 2 Regional Maxillofacial Unit University Hospital Aintree, Fazakerley, Liverpool L9 1AL, UK; 3 Medical Statistician, Regional Maxillofacial Unit University Hospital Aintree, Fazakerley, Liverpool L9 1AL, UK

**Keywords:** mouth neoplasm, head and neck cancer, survival, transfusion

## Abstract

The influence of perioperative blood transfusion in oral and oropharyngeal squamous cell carcinoma remains uncertain. It is believed that blood transfusion downregulates the immune system and may have an influence on cancer recurrence and survival. In all, 559 consecutive patients undergoing primary surgery for oral and oropharyngeal squamous cell carcinoma between 1992 and 2002 were included in this study. Known prognostic variables along with transfusion details were obtained from head and neck cancer and blood transfusion service databases, respectively. Adjusting for relevant prognostic factors in Cox regression, the hazard ratio for patients having 3 or more transfused units relative to those not transfused was 1.52 (95% confidence interval (CI) 0.93–2.47) for disease-specific and 1.52 (95% CI 1.05–2.22) for overall mortality. Blood transfusion of 3 or more units might confer a worse prognosis in patients undergoing primary surgery for oral and oropharyngeal squamous cell carcinoma. Therefore, every effort should be made to limit the amount of blood transfused to the minimum requirement.

Perioperative blood transfusions are reported to be related to cancer recurrence and reduced survival (Werther *et al*, 2001). Different underlying mechanisms have been proposed, and allogenic leucocytes in transfused blood have been suggested to contribute to this phenomenon (Jensen *et al*, 1992). The persistence of antigens in the recipient's circulation might also create an environment, which allows immune system downregulation. This possibility was supported by the evidence that allogenic blood transfusion enhances renal allograft survival; however, it has been hypothesised that blood transfusion-associated immune depression might be deleterious in cancer patients (Opelz *et al*, 1973; Gantt, 1981; Vamvakas and Blajchman, 2001). Although numerous clinical studies have addressed the question of perioperative blood transfusion and cancer recurrence and/or survival, the possibly harmful effect of allogenic blood transfusion on immunomodulation remains unresolved.

To date, there is relatively little published data on blood transfusion and outcome in oropharyngeal cancer (von Doersten *et al*, 1992; Sturgis *et al*, 1997; Taniguchi and Okura, 2003). In head and neck cancer, the effect of allogenic blood transfusion has been reported on relatively small numbers of patients with conflicting results (Table 1) (von Doersten *et al*, 1992; Woolley *et al*, 1992; Sturgis *et al*, 1997; Vamvakas and Blajchman, 2001; Taniguchi and Okura, 2003). Some authors have reported deleterious effects of blood transfusion (Jones and Weissler, 1990; Woolley *et al*, 1992; Taniguchi and Okura, 2003), while others could not confirm transfusion as an independent predictive factor in multivariate analysis (von Doersten *et al*, 1992; Schuller *et al*, 1994). A recent paper by Taniguchi and Okura (2003), specific to oral and oropharyngeal cancer, only showed the outcomes were worse with 3 or more units. This accumulating threshold effect of allogenic blood transfusion has already been seen with colonic and oesophageal cancers (Tartter, 1992; Langley *et al*, 2002). Hence, we undertook this study to test the hypothesis that perioperative blood transfusion has an adverse effect on survival of patients with oral and oropharyngeal cancer.

## MATERIALS AND METHODS

The study sample consisted of all consecutive patients undergoing primary surgery for previously untreated oral and oropharyngeal squamous cell carcinoma presenting to the Regional Maxillofacial Unit Liverpool, between the years 1992 and 2002. The Liverpool Oncology Head and Neck database was used to gather the clinical, demographic, surgical, pathological and outcome data. The data were downloaded into SPSS for further analyses.

Blood transfusion data were obtained from the centralised transfusion database of the Haematology Department of University Hospital Aintree. Perioperative haemoglobin levels were also collected if available from the computerised laboratory reporting system of the Haematology Department of University Hospital Aintree.

The Office of National Statistics supplied details of death certification for this patient cohort. Four clinicians independently attributed cause of death to oral cancer or other causes. In 10 cases (4% of deaths), there was a 50 : 50 judgement and further discussion between clinicians was required to reach a verdict.

### Statistical method

Association of factors with transfusion were tested by the *χ*^2^ test. Kaplan–Meier methods were used to estimate oral cancer disease-specific survival by patient groups and the log-rank test was used to compare survival curves. Cox regression methods were used to estimate the association of transfusion, and of transfusion with 3 or more units of blood, on survival after adjusting for covariates. The 95% confidence intervals for unadjusted and adjusted hazard ratios were obtained. Survival curve results were stratified by tumour p stage and adjusted Cox regression hazard ratios obtained. Stepwise Cox regression was used to find the best-fitting survival model from all available covariates and the linear prediction from this model was used to place patients into similarly sized ‘lower’ and higher' mortality risk groups. Survival curves by transfusion status were compared within these risk groups, and hazard ratios adjusted for the prognostic factors were also computed. The cutoff at 3 units of transfused blood was chosen to match the paper of Taniguchi and Okura (2003).

The preoperative haemoglobin cutoff value of 10 g dl^−1^ was set according to existing transfusion guidelines. Preoperation haemoglobin levels were routinely recorded from 1997 and were not included in the main prognostic analyses from 1992, but were included in separate analyses of the data from 1999 to 2002, analyses specifically undertaken to assess the situation following the adoption of leuco-depletion of blood in Aintree Hospitals from January 1999.

## RESULTS

The cohort comprised 559 patients undergoing primary surgery from 1992 to 2002 for previously untreated oral and oropharyngeal squamous cell carcinoma. Almost all (96%, 1342 of 1397) transfused units of blood during this time were for 437 patients having free-flap surgery. During the operation, 77% (337 of 437) were transfused with blood and 68% (223 of 330) of these received 3 or more units of blood, units unknown for seven patients. Transfusion rates *per se*, and rates for transfusions with 3 or more units, were highest for free-flap surgery patients with more advanced stages of disease, when soft-tissue margins were involved, with perineural invasion, for segmental resection and for composite-flap surgery (Table 2).

**Figure 1:**
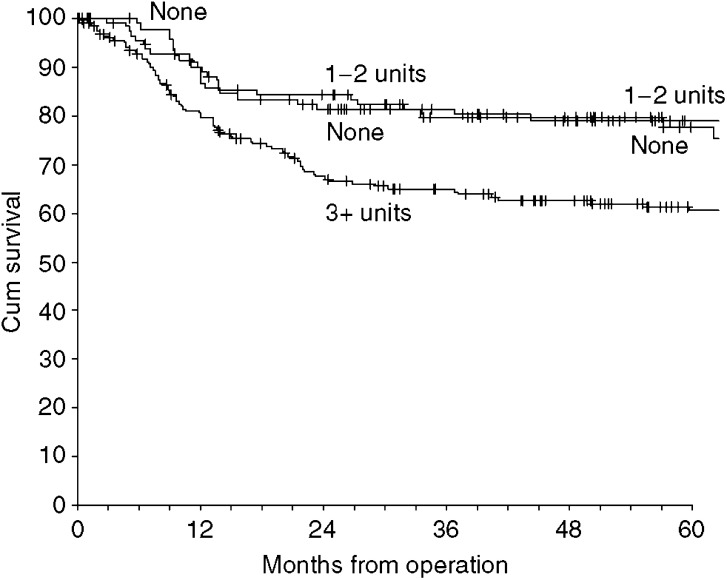
Kaplan–Meier disease-specific survival by whether patients had no blood transfusion, were transfused with 1–2 units or transfused with 3 or more units of blood.

The 5-year Kaplan–Meier estimate of oral cancer survival for patients receiving blood was 67% (s.e. 3%) as compared with 78% (s.e. 3%) for those not transfused. The relative risk of death (hazard ratio) from patients having a blood transfusion alone was estimated from Cox regression to be 1.59 (95% confidence interval (CI) 1.00–2.54). There was little difference in survival between those having no transfusions and those having 1 or 2 units transfused, but there was worse survival in those having 3 or more units transfused (Figure 1). The hazard ratio for patients with 3 or more transfused units was 1.93 (95% CI 1.20–3.11) relative to those not transfused.

**Figure 2:**
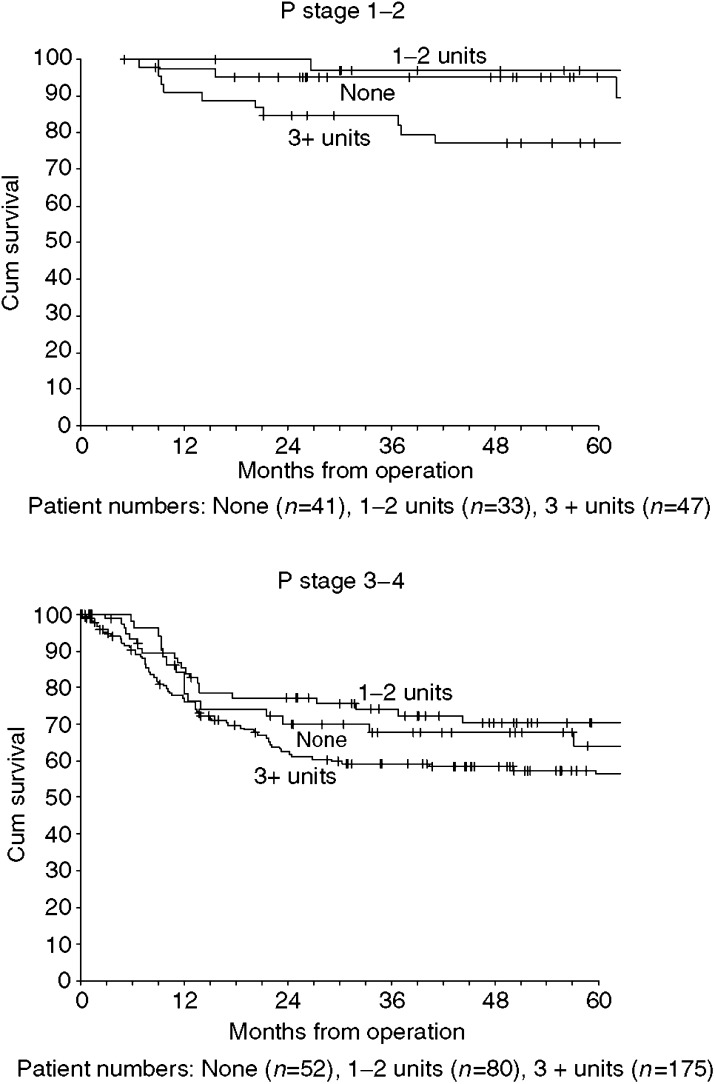
Kaplan–Meier disease-specific survival by blood transfusion and p stage.

**Figure 3:**
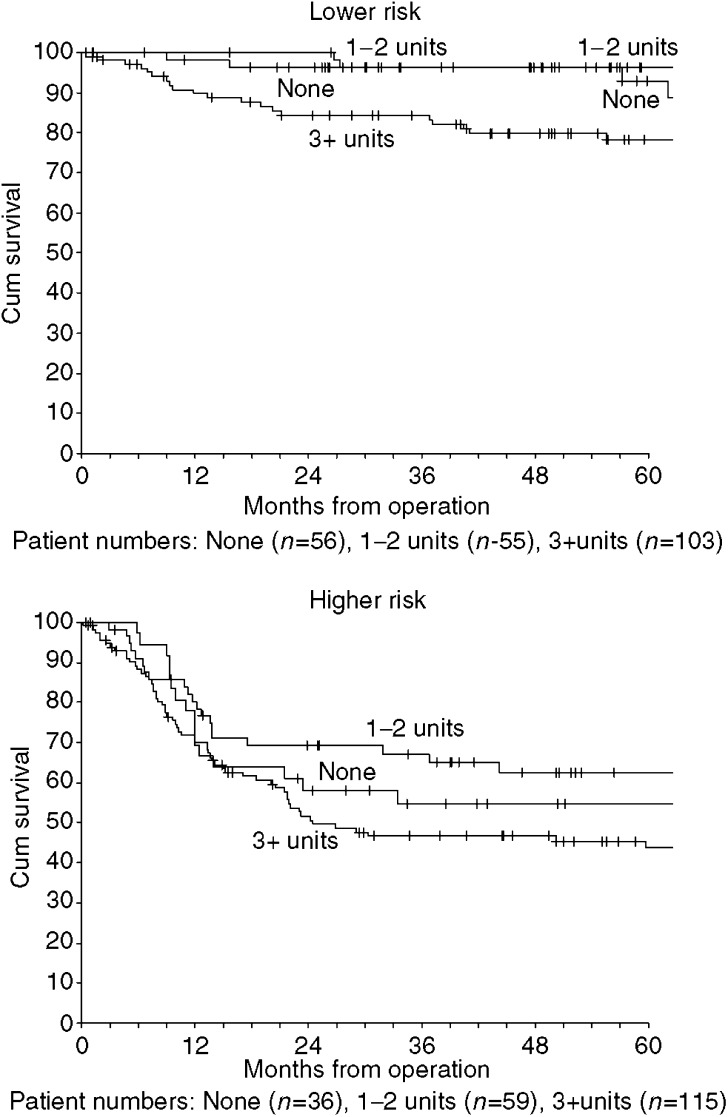
Kaplan–Meier disease-specific survival by blood transfusion and risk group (derived from Cox regression based on pN status and margins).

Many clinical and pathological factors were associated with disease-specific survival (Table 3) and there was inter-correlation between pathological factors. Patients having 3 or more units had the worse survival for both p1–2 and p3–4 stage tumours (Figure 2), and adjusting for p stage in Cox regression gave hazard ratios of 1.35 (95% CI 0.84–2.16) for transfusion and 1.56 (95% CI 0.96–2.52) for transfusion with 3 or more units. When all factors (of Table 3) were entered into a stepwise Cox regression (*P*<0.01 for entry), the two predictors selected were pN status and margins. The linear prediction from the regression model was used to place patients into similarly sized lower and higher risk groups. In effect, the lower risk group comprised 221 patients either with clear margins and pN0/pN1 or with close margins and pN0. Within each risk group, patients with 3 or more transfused units had the worst survival (Figure 3). Adjusting for pN status and margins in Cox regression gave a hazard ratio of 1.29 (95% CI 0.80–2.08) for transfusion and 1.52 (95% CI 0.93–2.47) for transfusion with 3 or more units.

All-cause mortality rates for patients receiving blood was 48% (s.e. 3%) as compared with 65% (s.e. 5%) for those not transfused. We found the same univariate predictors of all-cause mortality as for disease specific (data not shown). Cox regression gave unadjusted hazard ratios of 1.61 for transfusion and 1.84 for transfusion of 3 or more units, and adjustment for pN status, margins, age and perineural invasion (independent predictors at *P*<0.01) gave hazard ratios of 1.35 (95% CI 0.94–1.93) for transfusion and 1.52 (95% CI 1.05–2.22) for transfusion with 3 or more units.

**Figure 4:**
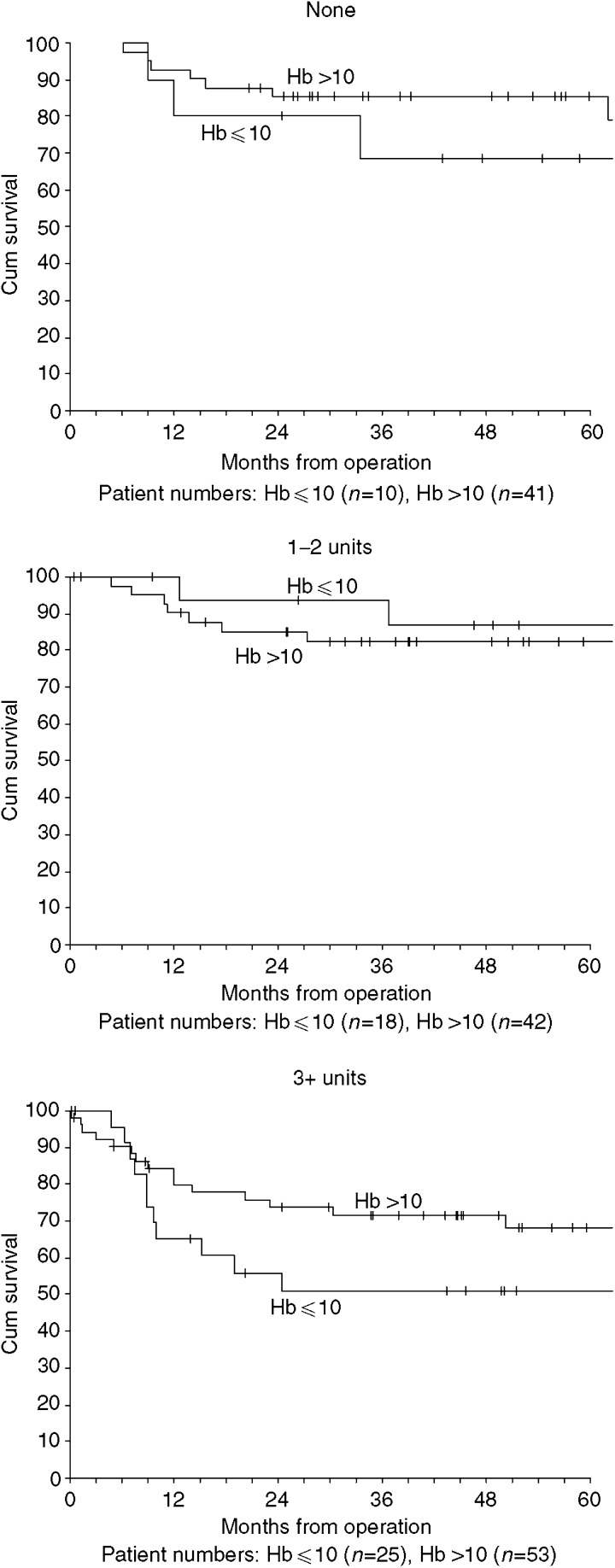
Kaplan–Meier disease-specific survival by blood transfusion and preoperation haemoglobin level.

Preoperation haemoglobin levels were routinely recorded from 1997 and were known for 81% (190 of 234). Transfusion rates were 81% (43 of 53) for patients with haemoglobin levels of 10 and under and 70% (95 of 136) for levels over 10. Rates for transfusion with 3 or more units were 47% (25 of 53) and 39% (53 of 136), respectively. The 5-year disease-specific survival rates were 65% (s.e. 7) and 78 (s.e. 4), *P*=0.19, and for all-cause survival, 44% (s.e. 7) and 60% (s.e. 5), *P*=0.07. The mean (s.d.) haemoglobin level was 12.2 (2.3) for patients not transfused, 11.9 (2.6) for those transfused 1–2 units and 11.3 (2.3) for those transfused 3 or more units. For patients transfused with 3 or more units, the observed disease-specific survival was worse for those with preoperative haemoglobin levels of ⩽10 (Figure 4), unadjusted hazards ratio 1.71 (95% CI 0.79–3.68) and after adjustment for pN status and margins 1.92 (95% CI 0.87–4.27).

From the beginning of January 1999, leuco-depletion of blood was introduced at University Hospital Aintree. In separate analyses of the data from 1999 for disease-specific survival, Cox regression methods gave unadjusted hazard ratios of 1.72 for transfusion and 2.22 for transfusion of 3 or more units, and after adjustment (pN status and margins), 1.34 (95% CI 0.63–2.85) for transfusion and 2.09 (95% CI 0.94–4.78) for 3 or more units. For all-cause survival, adjusted (pN status, margins, age and perineural invasion) ratios were 1.48 (95% CI 0.81–2.68) and 1.60 (95% CI 0.82–3.11).

Transfusion rates have fallen over time, 86% (239 of 279) before 1999 and 65% (98 of 151) from 1999 to 2002. Rates for transfusion with 3 or more units were 59% (165 of 279) and 38% (58 of 151), respectively. Before 1999, 5-year disease-specific survival was 68% (s.e. 3) and overall survival 51% (3), whereas from 1999 to 2002, 5-year disease-specific survival was 72% (s.e. 4) and overall survival 48% (5).

In the whole cohort, there was a higher rate of recurrence for patients transfused with 3 or more units (29%, 65 of 223) than for patients transfused with 1–2 units (19%, 22 of 114) and for patients not transfused (19%, 18 of 93) *P*=0.06 *χ*^2^ test. Local-only recurrence rates were 14% (31 of 222), 8% (nine of 114) and 6% (six of 93), respectively, *P*=0.08.

## DISCUSSION

In this large cohort of patients with oral and oropharyngeal cancer, the biggest influences on disease-specific and all-cause mortality were related to tumour pathology, resection margins and nodal status. Death certification was used to help give an indication as to the cause of death and help determine disease-specific survival. Although death certification in the absence of post mortem has its limitations owing to reporting errors, it has been shown to be of value in this cancer group (Leitner *et al*, 2001). Mortality was observed to be higher among patients receiving allogenic blood transfusion and higher still for those receiving 3 or more units. This association persisted after adjusting for confounding factors. These results were in keeping with those from previous authors, but were at the borderline of statistical significance (von Doersten *et al*, 1992; Woolley *et al*, 1992; Vamvakas and Blajchman, 2001; Taniguchi and Okura, 2003).

In the management of oral and oropharyngeal cancer prolonged surgical procedures involving tumour resection and reconstruction using free tissue transfer techniques will frequently result in significant intraoperative blood loss. Reported transfusion rates have varied widely from 32 to 81%, with more recent studies reporting lower transfusion rates (Taniguchi and Okura, 2003). This is in keeping with our experience, we observed a halving in transfusion rate from 88% in 1992 to 44% in 2002. It might be that more recent patients who have transfusion are more anaemic than patients earlier in the series. Hence, preoperative anaemia associated with transfusion may explain the enhanced hazard ratios from 1999 onwards. However, we cannot properly analyse the whole cohort, with respect to properative anaemia, owing to the lack of haemoglobin data prior to 1997. Survival is affected by several factors including malignancy stage, tumour resection margins and chronic systemic illness, all of which are confounding factors for allogenic blood transfusion (de Cassia Braga Ribeiro *et al*, 2003). A recent meta-analysis, which summarised findings of several observational studies, failed to show any significant outcome benefit between transfused and nontransfused patients (Vamvakas and Blajchman, 2001). Transfusion itself was associated with a 2.5-fold increase in mortality before adjustment for other factors, similar to our results presented above. In contrast to Vamvakas, however, our data suggest that transfusion of 3 or more units of blood is associated with a worse outcome in oropharyngeal cancer patients, with a reduction in survival and an increase in recurrence rates. Our data reinforce those of Taniguchi who observed an almost five-fold increase in the risk of death among patients transfused more than 3 units of blood compared to nontransfused patients (Taniguchi and Okura, 2003). Our data suggest that transfusion of 3 or more units of blood could confer a worse 5-year survival in oral cancer patients. Our patients were stratified into high- and low-risk groups, with this effect being observed in both groups (Figures 2 and 3). It seems, although more advanced disease carries a significantly worse prognosis, blood transfusion itself may accentuate this deleterious effect. To clearly answer the question whether blood transfusion affects survival, a randomised controlled trial is required. This would need to be multicentre and would involve withholding blood or blood products.

To date, clinical trials are inconclusive as to whether tumour recurrence rate is higher after transfusion in head and neck cancer (Jones and Weissler, 1990; Sturgis *et al*, 1997). Our study suggests that the local and systemic cancer recurrence rate is higher in those patients who were transfused with 3 or more units of blood. Given that the volume of blood transfused appears to be of prime importance in this worsened outcome, it is likely that the underlying cause is a dose-related immune suppression. The earliest reports of immunomodulation after blood transfusion in the 1970s were in the field of renal transplantation where transfusion before transplantation was accompanied with an improved allograft survival (Opelz *et al*, 1973; Opelz and Terasaki, 1978). Improved graft survival is associated with an increasing number of transfusions before transplantation, with a near maximal benefit after 5 units (Opelz and Terasaki, 1978). These data suggest that there may be an immunomodulatory threshold effect related to volume of blood transfused. In the context of cancer surgery, a similar finding has been demonstrated in that the adverse influence of blood transfusion on survival is seen only after larger transfusions for colorectal tumours, soft-tissue sarcomas and oesophageal cancers (Rosenberg *et al*, 1985; Tartter, 1992; Langley *et al*, 2002). Our results would appear to support the existence of a threshold effect, which has been demonstrated previously (Langley *et al*, 2002; Taniguchi and Okura, 2003).

Transfusion-related immunosuppression consists of a monocyte-mediated early phase and a later phase, which is characterised by increased suppressor T-cell activity. Both effects appear to be dependent on the number of transfusions (Dellinger and Anaya, 2004). Allogenic blood transfusion has been shown to impair natural killer cell function and also inhibit interleukin-2 production, both mechanisms crucial for normal antitumour activity (Dellinger and Anaya, 2004).

Although it is not entirely clear at molecular level which factors modulate the effects of allogenic blood transfusion in cancer surgery, there is good evidence that leucocytes in the blood mediate them (Jensen *et al*, 1992).

The patients in the current study did not have leukocyte-depleted transfusions until 1999, since when all allogenic blood for transfusion in our hospital has been leucocyte depleted. This is part of a nationwide policy in the United Kingdom aimed at minimising the possibility of variant Creutzfeldt–Jakob disease being passed through transfusion of blood orblood products. Although most of the possible detrimental effects of blood transfusion are thought to be mediated via the transfused leucocytes, we were unable to find any significant differences in the clinical outcome of patients before and after the leucocyte depletion policy was implemented.

It has been postulated that preoperative haemoglobin levels are independent predictors of survival in head and neck cancer (Macdonald and Hurman, 2004). In our series, we observed that patients who received 3 or more units of blood and had low preoperative haemoglobin had worse survival compared to those who had normal haemoglobin levels (Figure 4). This effect was less marked among those patients who received only 1–2 units of blood (Figure 4). This may suggest that patients whose disease is more advanced and manifests in severe preoperative anaemia mainly owing to reduced oral intake and dietary problems are much more vulnerable to the deleterious immunosuppressive effects of a larger allogenic blood transfusion.

Despite the limitations of the current study, including its retrospective nature, the results support previous work that demonstrated an adverse effect on survival of patients undergoing resection for oral carcinoma who receive an allogenic blood transfusion (Woolley *et al*, 1992; Taniguchi and Okura, 2003). Clinical trials with erythropoietin and acute normovolaemic haemodilution have shown promising results in the reduction of allogenic blood product requirements in maxillofacial surgery; however, the importance of the meticulous surgical technique cannot be overemphasised (Scott *et al*, 2002; Habler *et al*, 2004). Clinicians must be cognizant of the 3 unit threshold and should attempt to limit transfusion volume whenever it is safe to do so.

In conclusion, this study reaffirms the importance of completeness of resection and the importance of lymph node involvement in resection for oral carcinoma. In addition, it suggests that transfusion of 3 or more units of blood could adversely affect survival. Therefore, every effort should be made to limit the amount of blood transfused to the minimum requirement.

## Figures and Tables

**Table 1:** Effect of allogenic blood transfusion in other studies involving head and neck cancer patients

**Author (year)**	**Disease**	**No. in study**	**Transfusion rate (%)**	**Outcome**	**Results**
Taniguchi and Okura (2003)	Oral/oropharyngeal	105	32	5-year crude survival	*P*<0.01 (>3 units)
					
Moir *et al* (1999)	Larynx	165	36	Recurrence	*P*<0.04 (autologous *vs* heterologous)
Leon *et al* (1996)	Oral/oropharyngeal	269	32	Recurrence	No effect
	Larynx/hypopharynx				
Barra *et al* (1994)	Larynx/oral/oropharyngeal	207	73	5-year crude survival	*P*<0.05
Schuller *et al* (1994)	Larynx/oral/oropharyngeal	217	61	5-year crude survival	No effect
von Doersten *et al* (1992)	Larynx/paranasal/oral	104	49	Recurrence, infection	No effect
	Oropharyngeal				
Woolley *et al* (1992)	Larynx/hypopharynx	143	69	Recurrence	*P*<0.009
					
Alun-Jones *et al* (1991)	Larynx	69	55	5-year survival	*P*<0.05
					
Bock *et al* (1990)	Larynx/hypopharnyx	174	81	Recurrence, infection	No effect
				5-year crude survival	
Jones and Weissler (1990)	Larynx/oral/oropharyngeal	90	51	Recurrence	*P*<0.05

All studies reporting a significant difference do so for worse outcome associated with allogenic blood transfusion.

**Table 2:** Association of factors with blood transfusion for patients having free-flap surgery

		**Not transfused**		**Transfused <3 units**		**Transfused 3+ units**		
	**Patients**	**%**	***n***	**%**	***n***	**%**	***n***	***P*-value** ^*^
*Gender*								
Male	274	22	60	29	79	49	135	0.28
Female	156	21	33	22	35	56	88	
								
*Age (years)*								
<55	133	27	36	24	32	49	65	0.60
55–64	123	20	25	30	37	50	61	
65–74	115	18	21	26	30	56	64	
75+	59	19	11	25	15	56	33	
								
*Tumour site*								
Buccal	76	21	16	24	18	55	42	0.11
Lower gum	47	13	6	32	15	55	26	
Tongue (ant. 2/3)	87	28	24	32	28	40	35	
Floor of mouth	129	26	33	26	33	49	63	
Other	91	15	14	22	20	63	57	
								
*Type of flap*								
Soft	309	24	74	29	91	47	144	0.003
Composite	119	15	18	19	22	66	79	
								
*ASA*								
I	105	29	30	28	29	44	46	0.23
II	208	20	42	25	53	54	113	
III (*n*=77), IV(*n*=2)	79	16	13	27	21	57	45	
Unknown	38	21	8	29	11	50	19	
								
*Adjuvant radiotherapy*								
No	214	26	55	27	57	48	102	0.10
Yes	216	18	38	26	57	56	121	
								
*Mandibular resection*								
Nil	230	26	60	28	64	46	106	<0.001
Rim	73	22	16	37	27	41	30	
Segment	127	13	17	18	23	69	87	
								
*ECS*								
No ECS	313	22	69	27	83	51	161	0.94
ECS	117	21	24	27	31	53	62	
								
*Positive nodes*								
No	230	24	56	27	61	49	113	0.30
Yes	200	19	37	27	53	55	110	
								
*Margins*								
Clear >5 mm	187	27	50	27	51	46	86	0.001
Close <5 mm	147	25	37	24	35	51	75	
Involved	93	5	5	30	28	65	60	
								
*pT stage*								
Tis, 1–2	218	31	68	31	68	38	82	<0.001
3–4	210	12	25	21	45	67	140	
								
*pN*								
0, no neck	223	24	53	26	59	50	111	0.83
1	78	21	16	26	20	54	42	
2–3	129	19	24	27	35	54	70	
								
*P stage*								
1–2	121	34	41	27	33	39	47	<0.001
3–4	307	17	52	26	80	57	175	
								
*Tumour differentiation*								
Poor	55	11	6	20	11	69	38	0.04
Moderate	274	23	63	29	80	48	131	
Well	99	23	23	22	22	55	54	
								
*Pattern of invasion*								
Favourable	104	21	22	31	32	48	50	0.46
Unfavourable	323	22	70	25	80	54	173	
								
*Perineural invasion*								
No	304	25	77	27	81	48	146	0.009
Yes	126	13	16	26	33	61	77	
								
*Vascular invasion*								
No	289	24	69	26	75	50	145	0.27
Yes	141	17	24	27	39	55	78	

ASA=American Society of Anesthesiologists; ECS=extracapsular spread. ^*^χ^2^ test. Note the table excludes seven patients who had transfusions, but the number of units was unknown. T and N stage: Tis is *in situ*, 1 is 2 cm or less, 2 is more than 2 cm upto 4 cm, 3 is more than 4 cm up to 6 cm, 4 is greater than 6 cm or involving adjacent structures. Nodal stage: N0: No evidence of regional lymph node metastasis. N1: Metastasis in a single ipsilateral (same side) lymph node, 3 cm or less in size. N2: Metastasis in a single ipsilateral (same side) lymph node more than 3 cm but not more than 6 cm in greatest dimension, or metastasis in multiple ipsilateral (same side) lymph nodes, none more than 6 cm in greatest dimension, or metastasis in bilateral (both) or contralateral (opposite side) lymph nodes, none more than 6 cm in greatest dimension. N3: Metastasis in a lymph node more than 6 cm in greatest dimension. P stage is a combination of T and N status.

**Table 3:** Association of factors with disease-specific survival for patients having free-flap surgery

		**Disease specific**		
	**Patients**	**2 years**	**5 years**	***P*-value** ^*^
*Blood transfusion*				
No	93	81	79	0.001
<3 units	114	84	79	
3+ units	223	67	61	
				
*Gender*				
Male	279	76	71	0.29
Female	158	72	65	
				
*Age (years)*				
<55	137	80	76	0.008
55–64	125	73	66	
65–74	115	77	75	
75+	60	58	50	
				
*Tumour site*				
Buccal	76	74	69	0.51
Lower gum	47	79	74	
Tongue (ant. 2/3)	91	71	67	
Floor of mouth	132	78	73	
Other	91	72	64	
				
*ASA*				
I	109	81	79	0.07
II	210	74	66	
III/IV	80	71	64	
Unknown	38	68	62	
				
*Type of flap*				
Soft	316	76	71	0.29
Composite	119	71	66	
				
*Adjuvant radiotherapy*				
No	220	81	77	0.002
Yes	217	69	62	
				
*Mandibular resection*				
Nil	237	77	73	0.11
Rim	73	79	70	
Segment	127	68	63	
				
*ECS*				
No ECS	320	83	77	<0.001
ECS	117	52	46	
				
*Positive nodes*				
No	237	86	81	<0.001
Yes	200	61	55	
				
*Margins*				
Clear >5 mm	192	89	84	<0.001
Close <5 mm	149	71	64	
Involved	93	50	46	
				
*pT stage*				
Tis, 1–2	224	82	77	<0.001
3–4	211	66	61	
				
*pN*				
0, no neck	230	87	82	<0.001
1	78	73	68	
2–3	129	53	47	
				
*P stage*				
1–2	127	90	86	<0.001
3–4	308	68	62	
				
*Tumour differentiation*				
Poor	55	58	52	<0.001
Moderate	279	72	66	
Well	100	89	87	
				
*Pattern of invasion*				
Favourable	105	91	88	<0.001
Unfavourable	329	69	63	
				
*Perineural invasion*				
No	307	80	74	<0.001
Yes	130	60	56	
				
*Vascular invasion*				
No	294	82	76	<0.001
Yes	143	60	54	

ASA=American Society of Anesthesiologists; ECS=extracapsular spread. ^*^Log-rank test. Table gives 2- and 5-year Kaplan–Meier survival rates.
